# The role of cell-free DNA measured by a fluorescent test in the management of isolated traumatic head injuries

**DOI:** 10.1186/1757-7241-22-21

**Published:** 2014-03-19

**Authors:** Gad Shaked, Amos Douvdevani, Silvia Yair, Alexander Zlotnik, David Czeiger

**Affiliations:** 1 Department of General Surgery, Trauma Unit, Soroka University Medical Center, 68 Wingate St., Beer Sheva 84101, Israel; 2 Department of Clinical Biochemistry, Soroka University Medical Center, Beer Sheva, Israel; 3 Department of Anesthesiology, Soroka University Medical Center, Beer Sheva, Israel; 4 Soroka University Medical Center and Ben-Gurion University, Beer Sheva, Israel

**Keywords:** Cell free DNA, Circulating DNA, Brain injury, Head trauma

## Abstract

**Background:**

Traumatic brain injury (TBI) is a major cause of death and disability. In this study a new method to measure cell free DNA (CFD) for the management of TBI is tested. Our hypothesis was that CFD concentrations correlate to the magnitude of brain damage, and may predict the outcome of injured patients.

**Methods:**

Twenty eight patients with isolated head injury were enrolled. Their demographic and clinical data were recorded. CFD levels were determined in patients' sera samples by a direct fluorescence method developed in our laboratory.

**Results:**

Mean admission CFD values were lower in patients with mild TBI compared to severe injury (760 ± 340 ng/ml vs. 1600 ± 2100 ng/ml, p = 0.03), and in patients with complete recovery upon discharge compared to patients with disabilities (680 ± 260 ng/ml vs. 2000 ± 2300 ng/ml, p = 0.003). Patients with high CFD values had a relative risk to require surgery of 1.5 (95% CI 0.83 to 2.9) a relative risk to have impaired outcome on discharge of 2.8 (95% CI 0.75 – 10), and a longer length of stay (12 ± 13 days vs. 3.4 ± 4.8 days, p = 0.02). CFD values did not correlate with CT scan based grading.

**Conclusions:**

CFD levels may be used as a marker to assess the severity of TBI and to predict the prognosis. Its use should be considered as an additional tool along with currently used methods or as a surrogate for them in limited resources environment.

## Background

Traumatic brain injury (TBI) remains a major cause of death and disability. TBI is presented as a heterogeneous array of pathologies and severities [1]. This creates a significant challenge to the health providers, who take care of head injured patients, when attempting to identify and classify the patients that may benefit from a certain management. Currently, the basic classification of TBI patients is based on neurological injury severity criteria with the Glasgow Coma Scale (GCS) being the most commonly used scale for prognostic and follow-up evaluations, and to enroll patients into clinical trials [2-5]. In recent years, the traditional link between GCS and outcome has been questioned, and published data suggest that the predictive value of GCS should be reconsidered [6]. The Marshall [7] and the Rotterdam [8] scores for CT findings are the best known classification systems based on pathoanatomic features of the injury. Despite becoming widely used and serving as pragmatic tools, the CT-based classifications are not free of limitations [9]. To overcome the inherited drawbacks of the aforementioned clinical and pathoanatomic methods the role of biomarkers has been studied with S100, neuron-specific enolase, and Hsp 70 being among the most widely investigated [10-12]. However, failure to demonstrate adequate sensitivity and specificity prevented their routine clinical use as diagnostic or prognostic tools. Accumulative data favored the potential use of circulating cell-free DNA (CFD) in the plasma or serum for diagnosis, prognosis, and monitoring of a variety of conditions such as infection, inflammation, trauma, in critically ill patients with respiratory insufficiency or pulmonary embolism, in patients with autoimmune diseases, sepsis and cancer [13-19]. Pioneering studies explored the potential role of CFD as a surrogate biomarker in the management of TBI. Campello et al. demonstrated that severe TBI is associated with elevated CFD and that persistent increased concentrations of CFD correlate with mortality [20]. Macher et al. also showed that severe TBI is associated with augmented CFD levels, and suggested that early (within 24 hours) CFD concentrations decrease predicts a better outcome [21]. Despite these initial promising results on the value of CFD measurement in TBI patients this scheme has not yet entered into clinical use. A major obstacle is the applicability of the methods used to measure CFD. The currently available research methods for CFD measurement are work-intensive and expensive, requiring DNA extraction and real-time polymerase chain reaction amplification with specific primers. We recently developed a convenient DNA assay applied directly to biologic samples. This assay uses the fluorochrome SYBR Gold (Invitrogen, Paisley, Scotland), which does not require prior processing of samples. The assay is simply performed by adding diluted fluorochrome to the samples and measurement of fluorescence. The assay was proved to be accurate, sensitive, reproducible, cheap and rapid [22]. In a previous study, researchers from our institution found that CFD levels correlated with brain damage and with the neurological outcome after TBI in a rat model [23]. The aim of the present study was to evaluate this method for the identification of CFD in TBI patients, and whether it may serve as an additional diagnostic and prognostic tool capable to assist in the management of head injured patients. We hypothesized that CFD concentrations would associate with severity of injury, and that low or high levels could differentiate between good or bad outcome, respectively.

## Methods

### Setup

This is an observational prospective study that took place at the Soroka University Medical Center which is a tertiary care referral center and a level I trauma center in southern Israel. Patients sustaining brain injury are managed according to the Brain Trauma Foundation Guidelines [24].

### Study ethics

The research protocol was approved by the local Institutional Review Board of the hospital.

### Study population

Six hundred forty one patients sustaining head trauma were admitted to our hospital during the 1 year study period. Of this population 34 patients sustained blunt isolated head injury. Following the exclusion criteria, 28 patients were finally enrolled into the study.

### Exclusion criteria

Patients who demonstrated any other extra cranial injury with an Abbreviated Injury Score (AIS) over 1 were excluded from the study. Other exclusion criteria included age less than 18 years, pregnancy, malignancy, previous brain pathology, and admission later than 4 hours after the injury.

### Data collected

Patients' demographic, injury, clinical (including neurological and neuroimaging data), and treatment characteristics were collected. Specifically, we recorded the patients' age, gender, previous medical history, type (blunt or penetrating) and mechanism of injury, time from injury to blood sample withdraw, hypotension (admission systolic blood pressure <90 mmHg), list of injuries and Injury Severity Score (ISS), calculated probability of survival [the revised probability of survival (RPS) is a calculated percentage that is a prediction of the patient’s chance of surviving the trauma based on the age of the patient, the ISS, and the revised trauma score (RTS); it is automatically derived from our trauma registry software—Israeli Trauma Registry, ITR, version 2.2.2.14], admission GCS, a researcher blinded to the CFD results rated the admission brain CT scans according to the Marshall grading scale and to the Abbreviated Injury Scale (AIS), neurosurgical interventions (including intracranial pressure monitoring device placement, craniotomy for hematoma evacuation and decompressive craniectomy), length of stay in the intensive care unit and total hospitalization time, time on ventilator, use of vasopressors and inotropic medications, secondary complications: acute lung injury, systemic inflammatory response syndrome (SIRS) and sepsis, outcome measures included survival, discharge destination, and Glasgow Outcome Score (GOS) at hospital discharge. The Glasgow Outcome Score provides an objective assessment of the recovery of brain damaged patients in five categories ranging from minor neurological deficit to death [25]. Patient blood samples were obtained upon admission to the emergency department during the initial evaluation of the patients, and prior to any neurosurgical intervention took place. CFD levels were quantified by the fluorochrome SYBR® Gold assay which can be applied directly on biological fluids and was previously described to be in good correlation with the gold standard assay QPCR [22]. The treating teams were blinded to the results of the CFD assays.

### Statistical analysis

The mean CFD concentration of the TBI patients was compared to that of 30 healthy volunteers whose gender distribution and mean age were not different from the study cohort. Statistical analysis was performed with GraphPad Prism software (version 5.01; San Diego, CA). Statistically significant differences between 2 groups were tested by using the *t* test or the nonparametric Mann–Whitney test. Results are presented as mean ± SD. Categorical variables were analyzed by the Fisher's exact test. Significance was considered with p ≤ 0.05.

## Results

### Participant characteristics

A total of 28 patients were enrolled in the study. There were 23 (82%) males and 5 (18%) females. The median age of patients was 49 (range 18–91). All patients sustained blunt trauma. Table 1 shows patients demographic and clinical characteristics.

**Table 1:** Patients` demographics and clinical characteristics

**Age, year**	
Median (range)	49 (18-91)
Gender, n	
Male/female, n	23/5
Initial ^+^GCS, n	
≥14/≤13	14/14
Head ^*^AIS, n	
0-2/3-5	10/18
Types of cranial injury, n	
○ Epidural hematoma and/or Subdural hematoma	13
○ Subarachnoid hemorrhage	10
○ Intracerebral hemorrhage	5
○ Diffuse axonal injury	2
Craniotomies, n	8
^¤^LOS in ^∞^ICU, day	
Median (range)	1 (0-45)
In-hospital LOS, day	
Median (range)	4 (1-45)
^^^GOS at discharge from hospital, n (%)	
Dead	3 (11)
Vegetative state	0 (0)
Severely disabled	2 (7)
Moderately disabled	6 (21)
Good recovery	17 (61)

The table shows demographic and clinical characteristics of the patients enrolled in the study (^+^GCS = Glasgow coma score; ^*^AIS = Abbreviated injury score; ^¤^LOS = Length of stay; ^∞^ICU = Intensive care unit; ^^^GOS = Glasgow coma score).

**Figure 1:**
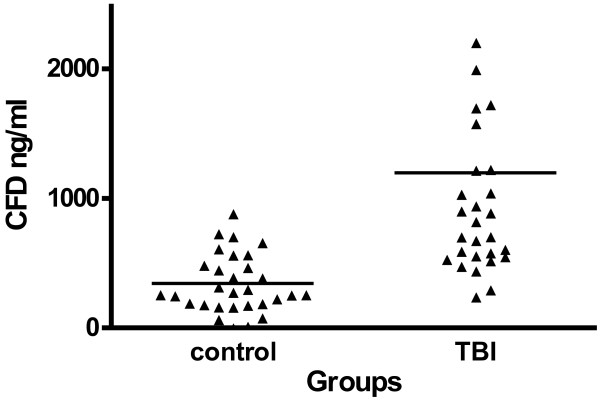
**CFD concentrations were determined in the sera of TBI patients and compared to CFD concentrations of healthy subjects.** The mean level of the TBI group was significantly higher than that of the control group (p = 0.0001).

The CFD concentrations of the patients were significantly higher than those found in the control group as demonstrated in Figure 1 (1200 ± 1600 ng/ml vs. 340 ± 230 ng/ml, p = 0.0001).

### Mortality

There were 3 patients (11%) who died in this series. These patients had severe head injury with low GCS (3,7,3), low RPS (27.5%, 44.7%, 20%), and extremely high CFD values (1925 ng/ml, 2203 ng/ml, 8787 ng/ml). The mean CFD concentration of the dead patients was significantly higher than that of the survivors (4305 ± 3884 ng/ml vs. 829 ± 404.9 ng/ml, p = 0.006).

### Clinical-anatomical grading

Comparison of the 14 patients with GCS of 14 or 15 with the 14 patients with GCS 13 – 3 demonstrated that the mean CFD concentration in the mild TBI group was significantly lower than in the group of severe TBI; 760 ± 340 ng/ml vs. 1600 ± 2100 ng/ml, respectively (p = 0.03).

We applied two CT scan based pathoanatomic classification schemes. We found no significant difference between the mean CFD concentrations of patients with Marshall 1 compared to Marshall ≥ 2, 1200 ± 750 ng/ml vs. 2200 ± 1000 ng/ml, p = 0.53. There was also no difference of mean CFD levels between the AIS group of 0 – 2 and AIS ≥ 3, 400 ± 1900 ng/ml vs. 750 ± 2500 ng/ml, p = 0.40.

### Outcomes

**Figure 2:**
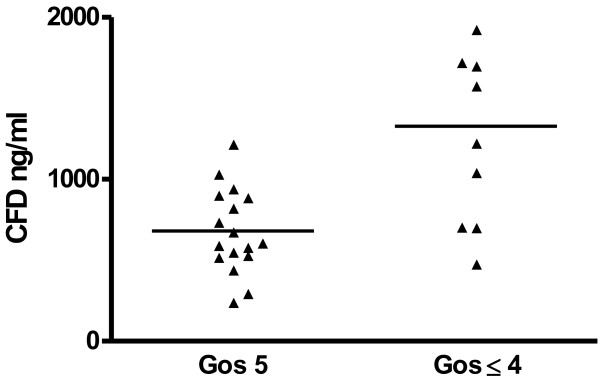
**The main outcome parameter was GOS.** The mean CFD concentration in the full recovery group (GOS 5) was significantly lower than that of the less favorable outcome group (GOS 1–4), p = 0.003.

The GOS was used as the main outcome measure. The mean CFD concentrations were significantly higher in the group of GOS ≤ 4, 2000 ± 2300 ng/ml vs. 680 ± 260 ng/ml in the group of GOS 5, p = 0.003 (Figure 2).

**Figure 3:**
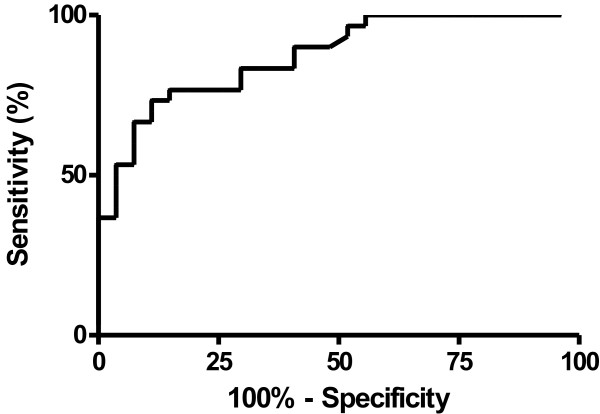
**Receiver operating characteristic curve analysis of CFD concentrations of TBI patient group compared with control group.** Calculated area under the curve = 0.872 (95% CI 0.784- 0.961).

To determine the upper CFD cutoff levels, we performed a receiver operating characteristic curve (ROC, Figure 3) analysis and found an area under the curve of 0.872 (95% CI0.784- 0.961). The maximal likelihood ratio (2.21) for sensitivities of 90% was 700 ng/ml, and this value was chosen as the upper CFD cutoff. Using the cut-off level of CFD 700 ng/ml the relative risk to have a GOS < 5 on discharge from hospital was 2.8 (95% CI 0.75 - 10) when the CFD concentrations were high.

Using the same cut-off point, a relative risk of 1.5 (95% CI 0.83 - 2.9) for surgical procedure was found with high CFD concentrations.

The in-hospital length of stay was significantly longer for patients with CFD > 700 ng/ml, 12 ± 13 days vs. 3.4 ± 4.8 days when CFD < 700 ng/ml, p = 0.02.

The power of CFD was analyzed to predict good recovery (GOS = 5) or an outcome with significant degree of disability (GOS < 5). The sensitivity and specificity were found to be 82% (9/11) and 59% (10/17), respectively, when the cut-off value of CFD was 700 ng/ml. In comparison, the sensitivity and specificity in this study for GCS above or below 14 as a measure to have an outcome of GOS 5 or GOS < 5 was 73% (8/11) and 65% (11/17), respectively. The negative and positive predictive values of CFD values alone were 83% (10/12) and 56% (9/16), respectively. In comparison, these proportions for GCS were 79% (11/14) and 57% (8/14), respectively. However, when we did the analysis by using CFD < 700 ng/ml *and* GCS ≥ 14 compared with CFD > 700 ng/ml *and* GCS ≤ 13 it was found that the sensitivity of the combined positive parameters when having GOS < 5 was 100% (6/6), and the negative predictive value of the combined negative parameters to predict GOS 5 was 100% (8/8). A specificity of 67% (8/12) of the combined parameters was higher than each of them alone, and so also for a positive predictive value of 60% (6/10). Interestingly, in the subgroup of patients with combined negative parameters and GOS 5 upon discharge none of the patients required surgical intervention to evacuate a cranial hematoma.

## Discussion

The main results of this study show that CFD levels are elevated in TBI patients with GCS ≤ 13, and in patients with worse outcome as reflected by mortality, poor GOS, prolonged length of stay in the hospital, and with increased risk to require a surgical intervention.

All patients included in this study suffered isolated TBI. This is in contrast with previous studies that investigated the significance of CFD in the management of TBI in which most of the enrolled patients suffered associated extra cranial injuries [20,21]. Plasma DNA concentrations increase early after injury and are higher in patients with severe injuries and in those who develop organ failure. Increased plasma DNA persists for days after injuries, especially in patients with multiple organ dysfunction syndrome [14,26]. Therefore, we excluded in the present study any significant extra cranial injuries in order to enable a better understanding of the impact of brain injury on CFD. We assume that CFD levels measured early after isolated TBI reflect only the brain tissue damage.

In our series 3 (11%) patients died. All the three patients that succumbed demonstrated remarkably high CFD values. This finding is in agreement with other reports that suggested that high persistent CFD levels may predict fatal outcome [14,20,21,27]. The relatively small number of dead led us to use the GOS which includes mortality as our main outcome measure. GOS actually reflects the combined severity of injury and the quality of care given to the patients. Since all the patients in this study were treated in a single center using standard protocols the GOS can be used as a reliable end point measure. The mean CFD values of patients with GOS 5 (good recovery) were significantly lower compared with the other grades of GOS, and high values (in this study 700 ng/ml was determined as the cut-off point) represented a 2.8 relative risk for incomplete recovery.

To date, most clinicians base their prognostication and initial decision making process on the neurologic injury severity criteria defined by the GCS and on the CT scan appearance of the brain injury. In this study we showed that the mean value of admission CFD concentrations in mild head injury (GCS >13) was significantly lower than that of moderate and severe TBI. Albeit its time-acknowledged role in the management of head injured patients there are many reported limitations with the use of this scale [28-31]. Our findings show that combining the CFD concentrations to the GCS results in a dramatic improvement of the prognostication. The negative predictive value of low CFD values plus GCS scores > 13 for good outcome (GOS 5) was 100%, and the sensitivity of positive combined results was 100%. These findings may be of utmost importance in the decision making process of emergency medicine providers working in austere environments lacking modern imaging modalities; This highly sensitive combination of tests can predict accurately a good outcome of patients probably without a need for surgical intervention. Our "mix and measure" technique requires equipment that can be operated by any hospital that has a chemistry laboratory. The cost per one test is less than $1. In the future it is possible to manufacture a small point-of-care measuring device.

It has been more than 3 decades since the CT scan has been introduced as the mainstay evaluation tool of neurotrauma patients [32]. Despite the usefulness of CT brain scan in clinical practice there is still uncertainty regarding the ability of CT based classification methods to predict outcome [33]. In the present study we used AIS and Marshall Classification to classify the findings in CT scans. There was no significant difference between CFD values of mild and severe grades of injury as determined by these schemes. Our explanation to this discrepancy is that CFD levels may better reflect the secondary damage to brain tissue than the CT scan. Primary injury refers to the immediate parenchymal damage occurring at the time of injury, while secondary damage refers to potentially avoidable damage that occurs at some time point after the primary insult. The second injury is caused by accompanying conditions to the trauma such as hypoxia, shock, hypercarbia, hyponatremia, seizures, high temperature, and brain edema of tissue surrounding the primary lesion. This conditions lead to physiologic derangements of brain parenchyma and to inevitable progressive cell damage. Another possible explanation is that even today the CT fails to accurately evaluate the magnitude of damage inflicted to the brain by diffuse axonal injury without mass lesion; however, massive cell death in this situation would result in high levels of CFD. On the other hand, a word of caution about how CFD (as well as other biomarkers) reflects the grade of injury severity should be made. Biomarkers may not be able to differentiate between a mild injury to a large volume of brain tissue from a severe injury involving a small area, or between two similar pathologies that are located in different parts of the brain in a way that may greatly affect the outcome. This concern was raised by an expert panel assembled by the National Institute of Neurological Disorders and Stroke in 2007 [34].

Limitations of the study include: A relatively small cohort of patients; the inability to compare our results to other studies since we use a different method to measure CFD concentrations; we did not look into the possible effect of prehospital interventions on CFD levels; the applicability to clinical situations in which patients sustain multiple injuries.

## Conclusions

The results of this study add to previous reports about the promising role of biomarkers and particularly CFD in the evaluation of TBI patients. CFD can increase the accuracy of existing tools such as GCS and imaging technology to differentiate mild TBI from more severe injuries. The technique we developed in our laboratory to determine CFD concentrations is simple, rapid, and non-expensive. Thus, it seems to be potentially appealing to apply in austere conditions where rapid transfer of patients to higher levels of care is considered.

## Competing interests

This work was supported by the “Dr. Montague Robin Fleisher Kidney Transplant Unit Fund”. The fund was not involved in any way in the study. Amos Douvdevani submitted a US Patent Application No. 13/659,439 “Assay for Detecting Circulating Free Nucleic Acids”. All other authors deny any other financial or personal relationships that might be considered as conflict of interest.

## Authors’ contributions

GS participated in the design of the study and in writing the manuscript. AD developed the laboratory method used in this study, supplied technical assistance, and took part in writing the manuscript. SY was involved in the enrollment of patients, the follow-up, coordination, and data collection. AZ drafted the manuscript and participated in the analysis. DC was involved in the design of the study, made the statistical analysis, and assisted in writing the manuscript. All authors read and approved the final version of the manuscript.
